# An outbreak of Rift Valley fever among peri-urban dairy cattle in northern Tanzania

**DOI:** 10.1093/trstmh/trac076

**Published:** 2022-08-30

**Authors:** William A de Glanville, Kathryn J Allan, James M Nyarobi, Kate M Thomas, Felix Lankester, Tito J Kibona, John R Claxton, Benjamin Brennan, Ryan W Carter, John A Crump, Jo E B Halliday, Georgia Ladbury, Blandina T Mmbaga, Furaha Mramba, Obed M Nyasebwa, Matthew P Rubach, Melinda K Rostal, Paul Sanka, Emmanuel S Swai, Agnieszka M Szemiel, Brian J Willett, Sarah Cleaveland

**Affiliations:** Institute of Biodiversity, Animal Health and Comparative Medicine, University of Glasgow, Glasgow, G12 8QQ, UK; University of Global Health Equity, Kigali 6955, Rwanda; Institute of Biodiversity, Animal Health and Comparative Medicine, University of Glasgow, Glasgow, G12 8QQ, UK; School of Veterinary Medicine, University of Glasgow, Glasgow G61 1QH, UK; Institute of Biodiversity, Animal Health and Comparative Medicine, University of Glasgow, Glasgow, G12 8QQ, UK; Nelson Mandela African Institution of Science and Technology, Arusha 255, Tanzania; Centre for International Health, University of Otago, Dunedin 9054, New Zealand; Kilimanjaro Clinical Research Institute, Moshi 2236, Tanzania; Paul G. Allen School for Global Health, Washington State University, Pullman, WA 99164, USA; Global Animal Health Tanzania, Arusha 1642, Tanzania; Nelson Mandela African Institution of Science and Technology, Arusha 255, Tanzania; Institute of Biodiversity, Animal Health and Comparative Medicine, University of Glasgow, Glasgow, G12 8QQ, UK; MRC-University of Glasgow Centre for Virus Research, University of Glasgow, Glasgow, G61 1QH, UK; Institute of Biodiversity, Animal Health and Comparative Medicine, University of Glasgow, Glasgow, G12 8QQ, UK; Centre for International Health, University of Otago, Dunedin 9054, New Zealand; Division of Infectious Diseases and International Health, Duke University Medical Center, Durham, NC 27710, USA; Duke Global Health Institute, Duke University, Durham, NC 27710, USA; Kilimanjaro Christian Medical University College, Tumaini University, Moshi 3010, Tanzania; Institute of Biodiversity, Animal Health and Comparative Medicine, University of Glasgow, Glasgow, G12 8QQ, UK; Institute of Biodiversity, Animal Health and Comparative Medicine, University of Glasgow, Glasgow, G12 8QQ, UK; Kilimanjaro Clinical Research Institute, Moshi 2236, Tanzania; Division of Infectious Diseases and International Health, Duke University Medical Center, Durham, NC 27710, USA; Kilimanjaro Christian Medical University College, Tumaini University, Moshi 3010, Tanzania; Tanzania Veterinary Laboratory Agency, Dar es Salaam 9254, Tanzania; Ministry of Livestock and Fisheries, Dodoma 40487, Tanzania; Division of Infectious Diseases and International Health, Duke University Medical Center, Durham, NC 27710, USA; Duke Global Health Institute, Duke University, Durham, NC 27710, USA; Programme in Emerging Infectious Diseases, Duke-National University of Singapore, Singapore 169857, Singapore; Institute of Biodiversity, Animal Health and Comparative Medicine, University of Glasgow, Glasgow, G12 8QQ, UK; EcoHealth Alliance, New York, NY 10018, USA; Tanzania Veterinary Laboratory Agency, Dar es Salaam 9254, Tanzania; Ministry of Livestock and Fisheries, Dodoma 40487, Tanzania; MRC-University of Glasgow Centre for Virus Research, University of Glasgow, Glasgow, G61 1QH, UK; MRC-University of Glasgow Centre for Virus Research, University of Glasgow, Glasgow, G61 1QH, UK; Institute of Biodiversity, Animal Health and Comparative Medicine, University of Glasgow, Glasgow, G12 8QQ, UK

**Keywords:** Africa, food microbiology, milk, population surveillance, Rift Valley fever, zoonoses

## Abstract

**Background:**

Human and animal cases of Rift Valley fever (RVF) are typically only reported during large outbreaks. The occurrence of RVF cases that go undetected by national surveillance systems in the period between these outbreaks is considered likely. The last reported cases of RVF in Tanzania occurred during a large outbreak in 2007–2008.

**Methods:**

Samples collected between 2017 and 2019 from livestock suffering abortion across northern Tanzania were retrospectively tested for evidence of RVF virus infection using serology and reverse transcription quantitative polymerase chain reaction (RT-qPCR).

**Results:**

A total of 14 RVF-associated cattle abortions were identified among dairy cattle in a peri-urban area surrounding the town of Moshi. RVF cases occurred from May to August 2018 and were considered to represent an undetected, small-scale RVF outbreak. Milk samples from 3 of 14 cases (21%) were found to be RT-qPCR positive. Genotyping revealed circulation of RVF viruses from two distinct lineages.

**Conclusions:**

RVF outbreaks can occur more often in endemic settings than would be expected on the basis of detection by national surveillance. The occurrence of RVF cases among peri-urban dairy cattle and evidence for viral shedding in milk, also highlights potentially emerging risks for RVF associated with increasing urban and peri-urban livestock populations.

## Introduction

Rift Valley fever (RVF) is a mosquito-borne disease caused by the Rift Valley fever virus (RVFV) that affects people and animals across Africa and the Arabian Peninsula. Previous RVF outbreaks have been major public health emergencies in affected countries and the disease is considered to be a priority for research and intervention by the World Health Organization (WHO).^1^ Ruminant livestock are highly susceptible to RVFV, with disease in cattle, goats and sheep associated with abortion and mortality in young animals.^2^

The epidemiology of RVF in East Africa is characterised by infrequent epidemics that are triggered by the emergence of large numbers of floodwater mosquitoes following periods of unusually heavy rainfall.^3^ In Tanzania, RVF epidemics have been reported every 10–15 y, with the last human or animal cases identified in the country in the year 2007.^4^ While clinical disease in people and animals is typically not reported outside epidemics in most countries in East Africa, an increasing number of serological surveys provide evidence for regular circulation of RVFV during this interepidemic period.^2,5,6^

Surveillance for RVF in most endemic countries, including Tanzania, is passive, requiring the reporting of suspicious illness in people or livestock. However, detection and confirmation of RVF cases during outbreaks is likely to be challenging for several reasons. In people and animals, presenting symptoms and signs of RVF are typically non-specific.^2^ Evidence-based thresholds for reporting and investigating unusually high rates of abortion or mortality in livestock have also generally not been established. Where suspect human or animal cases are reported, confirmation requires RVF-specific laboratory tests, which can have limited availability in endemic countries. These issues are compounded by medical and veterinary infrastructures being particularly weak in the grassland areas that tend to be at highest risk for RVF.^3^ Such infrastructure can be further weakened during periods of flooding, which are also the periods when risk of RVFV transmission is greatest.^7^ Passive surveillance for RVF in these circumstances is likely to have very low sensitivity for detecting disease events, particularly events with small numbers of cases or with limited geographic range.

Given regular circulation of RVFV and expected low sensitivity of current surveillance for disease detection, the occurrence of small-scale outbreaks with undetected human and animal RVFV infections during periods of heavy rainfall is likely.^2,7^ Such disease events could occur regularly: in the period 2013–2019, meteorological models predicted multiple areas of northern Tanzania to be at high risk for RVF outbreaks in all years except 2014.^8^

Here we describe a previously unreported outbreak of RVF among dairy cattle near the town of Moshi, Tanzania that occurred in 2018. Infections were detected retrospectively as a result of RVF testing performed on samples collected as part of a research study investigating the infectious aetiology of cattle, goat and sheep abortions across northern Tanzania. The outbreak provides an example of the type of small-scale RVF disease event that may go undetected by national animal health surveillance systems.

## Methods

### Prospective cohort study of livestock abortion

A prospective cohort study of livestock abortions was conducted from November 2017 through October 2019 in three administrative regions of northern Tanzania. This study and its principal findings are described in detail elsewhere.^9^ Briefly, the study population included all livestock-keeping households in 13 wards in Arusha, Manyara and Kilimanjaro Regions. Livestock keepers in these regions have been classified as engaging in a mixture of agropastoral, pastoral and smallholder-based livelihoods.^10^

Each study ward was served by a livestock field officer (LFO), a government-employed veterinary technician providing basic veterinary services. Community meetings were held in study wards and livestock keepers were asked to report any cattle, goat and sheep abortions to their LFO. Within 72 h of a reported abortion, the LFO collected blood, milk and vaginal swab samples from the aborting dam. Placental tissue samples and foetal surface swabs were also collected where available. Tissue, milk and swab samples were preserved in DNA/RNA shield (Zymo Research, Irvine, CA, USA). Basic individual animal- and household-level data, including breed, service date, animal demographics and vaccination history, were recorded.

All households were revisited 4–6 weeks after the investigated abortion and a convalescent-phase blood sample was collected from the affected dam.

### RVF livestock abortion case definition

Based on World Organisation for Animal Health (OIE) guidelines,^11^ we used a combination of molecular and serological methods to identify RVF cases. A case of RVF-associated livestock abortion was defined as an abortion event with RVFV detected by one-step reverse transcription quantitative polymerase chain reaction (RT-qPCR) in any of the vaginal swab, foetal swab or placental tissues and evidence of RVFV antibodies by enzyme-linked immunosorbent assay (ELISA) on a blood sample collected on an acute or convalescent serum sample from the dam.^9^

RVF molecular and serological testing was performed retrospectively, with diagnoses made approximately 12 months after abortion events occurred. Households in which RVF cases were identified were revisited shortly after detection (i.e. around 12 months post-abortion) and livestock keepers informed of positive test results. During this third visit, a blood sample was collected from any animals that had been confirmed as RVF cases and were still present.

### Serological testing for RVFV

Sera were separated from clotted whole blood samples by centrifugation (≤1300 *g* for 10 min). Sera from acute, convalescent and 12-month blood samples were tested for RVFV immunoglobulin G antibodies using a multispecies competitive ELISA (ID Screen, IDVet, Paris, France).

### RT-qPCR

RNA was extracted from swab, tissue and milk samples using a RNeasy Mini Kit (Qiagen, Hilden, Germany) as described by Thomas et al.^9^ Details on extraction from milk samples are given in the supplementary materials. Detection of RVFV by RT-qPCR on extracted RNA was performed using published protocols.^12^ Briefly, a 94 base pair (bp) target of the RVFV G2 gene was amplified using the primer pair RVS and RVA and a fluorescent-labelled probe (RVP). A negative extraction control, negative PCR control (PCR grade water) and positive control (RNA extracted from cells experimentally infected with RVFV-MP12^13^) were included in each PCR run. Samples were tested in duplicate, with crossover threshold (Ct) values <40 on both wells considered positive.

### Genotyping of RVFV

RNA from positive RT-qPCR samples was shipped to the University of Glasgow for viral genotyping. An approximately 900-bp target of the S genome segment was amplified using previously described primers.^14^ Reactions were performed using the Qiagen OneStep RT-PCR Kit using 8 μL of extracted RNA in a final reaction volume of 25 μL. Initial cycling conditions were reverse transcription at 50°C for 60 min followed by an initial PCR activation step of 95°C for 15 min and then 45 cycles of 94°C for 1 min, 60°C for 1 min and 72°C for 1.5 min. A final extension step of 72°C for 10 min was performed. For samples that failed to amplify under these initial conditions, the RT-qPCR was repeated using an extended RT step of 90 minutes at 50°C to increase the amount of complementary DNA template available for amplification. A negative control (PCR grade water) and positive control (RVFV-MP12) were included in each run. PCR products were visualised by gel electrophoresis. For sequencing, PCR products from positive samples were purified using the QIAquick Gel Extraction Kit (Qiagen). Sanger sequencing was performed using both forward and reverse primers by Source Bioscience (Nottingham, UK) using deoxyguanosine triphosphate technology.

Molecular phylogenetic analysis was performed in MEGA 7.0.^15^ Sequences were aligned using the Clustal W algorithm.^15^ The evolutionary history was inferred using the maximum likelihood method based on the Kimura two-parameter model with a discrete gamma distribution.^16^ RVFV sequences were compared with sequences from a diverse set of RVFV strains through BLAST searches of the GenBank nucleotide database to identify the infecting haplotype.^17^ The tree with the highest log likelihood value was selected and drawn to scale with branch lengths measured in the number of substitutions per site. All positions containing gaps or missing data were deleted.

In our analysis, particular attention was paid to viral sequences that fell into the same haplotype clade as the live vaccine used in the region (Smithburn RVFV modified vaccine strain, GenBank accession number DQ380157), as this can induce abortions in pregnant animals.^18^ We therefore sought to rule out vaccination as a cause of abortion among animals meeting our case definition. For this, phylogenetic analysis was first performed to estimate the genetic distance between samples obtained from case animals and the reference Smithburn vaccine strain. Next, the nucleotide sequences from field samples were translated into amino acid sequences using National Center for Biotechnology Information (NCBI) BLAST tools,^19,20^ aligned and compared with reference amino acid sequences from the Smithburn vaccine strain (DQ380157). We determined whether nucleotide substitutions in field-derived viral strains were synonymous mutations, which could indicate a modified vaccine strain, or non-synonymous mutations, which would be more consistent with a wild-type virus infection.

## Results

### Prospective cohort study of livestock abortion

Samples were collected from 215 abortion events from November 2017 through October 2019.^9^ Of the investigated abortions, 71 (33%) involved cattle, 100 (47%) goats and 44 (21%) sheep. Additional details on the breeds of affected animals are given in the supplementary materials. Both RT-qPCR and acute or convalescent phase serological testing for RVF were performed on samples from 212 of 215 (99%) abortion events. The number of abortion events investigated per ward was highly variable, with a minimum of 1 and maximum of 84 abortion events investigated (Supplementary Table S1).

### Detection of RVF livestock abortion cases

**Figure 1.**
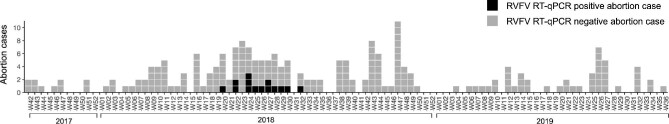
RVFV RT-qPCR status of livestock abortions in northern Tanzania by week, November 2017–October 2019.

**Figure 2.**
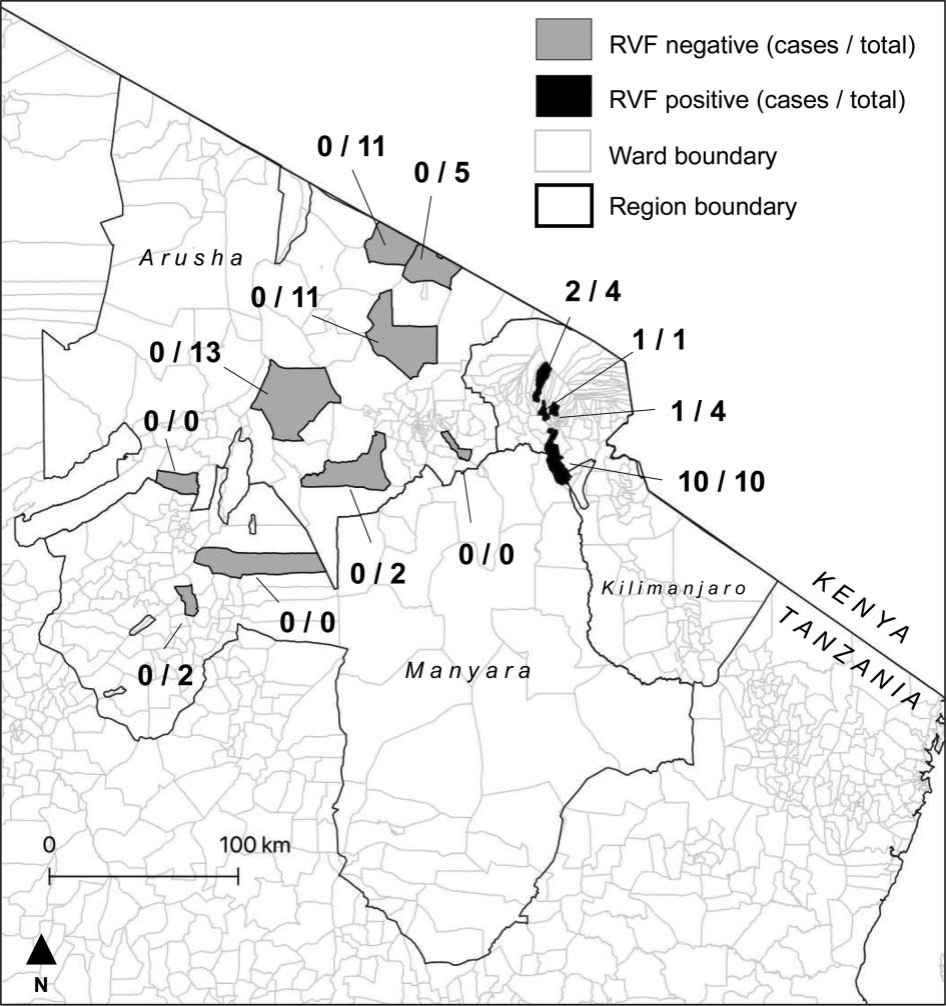
Location of wards included in the prospective cohort study of livestock abortion in northern Tanzania, 2017– 2019. The number of RVF cases identified out of total abortions investigated during the apparent outbreak period (16 May–11 August 2018) are shown.

A total of 14 abortion events met our case definition for RVF-associated abortion. All occurred in cattle between 16 May and 11 August 2018 in 14 households. These 14 cases represented 23% of the 63 abortion investigations conducted as part of the prospective cohort study over this time period (i.e. the apparent outbreak period) (Figure 1). Case animals were all found in four wards in three districts surrounding the town of Moshi in the Kilimanjaro Region (Figure 2). These were Arusha Chini and Kindi Wards in Moshi Rural District, Rau Ward in Moshi Municipality and Machame Mashariki Ward in Hai District.

Table 1 shows the samples collected from case animals and the results of RT-qPCR and serological testing performed. Further details on Ct values for RT-qPCR and percentage competition ELISA values are given in the Supplementary Tables S2 and S3. Milk samples and vaginal swabs were available from all 14 cases, of which 3 (21%) and 11 (79%) were positive by RT-qPCR, respectively. Ten (91%) of 11 foetal swabs and all 6 (100%) placental samples from confirmed cases were positive for RVFV by RT-qPCR. Of the 14 RVF abortion cases, 13 (93%) dams were seropositive on the basis of samples collected within 72 h of abortion and 1 (7%) case demonstrated seroconversion between acute and convalescent sera (Table 1). All seven case animals that could be sampled at 12 months post-abortion remained seropositive (Table 1 and Supplementary Table S2).

**Table 1.** Results of RT-qPCR and serological testing performed on samples from confirmed cattle RVF cases in northern Tanzania, May–July 2018

		RT-qPCR				Serology		
Abortion date (2018)	District	Milk	Vaginal swab	Placental sample	Foetal swab	Acute serum	Convalescent serum	12-month serum
16 May	Moshi Rural	−	+	NA	+^a^	−	+	+
29 May	Moshi Rural	+	+	NA	+	+	+	+
29 May	Moshi Rural	+	+	NA	+	+	+	+
12 June	Moshi Rural	−	+	NA	+	+	+	+
11 June	Moshi Rural	−	+	+	+	+	NA	+
17 June	Moshi Rural	+	−	+^b^	−	+	+	NA
24 June	Moshi Rural	−	−	+	+	+	+	NA
29 June	Moshi Rural	−	+	NA	NA	+	+	+
3 July	Moshi Municipality	−	+	NA	+^c^	+	+	+
3 July	Moshi Rural	−	+	NA	NA	+	NA	+
15 July	Hai	−	−	+	NA	+	+	NA
22 July	Moshi Rural	−	+	NA	+	+	+	+
26 July	Hai	−	+	+	+	+	+	+
11 August	Moshi Rural	−	+	+	NA	+	+	NA

+: positive sample; −: negative sample; NA: sample type that was not available.

Samples used for genotyping (individual identifiers): ^a^SEBI-051, ^b^SEBI-079, ^c^SEBI-094.

One cow that aborted in August 2018 in Hai District and one cow that aborted in July 2019 in Moshi Rural District were also seropositive for RVFV on acute serum samples (and the one available convalescent sample from these animals). Both animals were negative by RT-qPCR testing of milk, vaginal swabs and foetal swabs and so did not meet the case definition for RVF-associated abortion.

### Characteristics of RVF cases and affected households

Of the 14 RVF cattle, 1 (7%) was an indigenous breed, 7 (50%) were European dairy breeds and 6 (43%) were cross-breeds. The service date was known for 12 case animals, with abortions occurring at a median of 181 d of gestation (range 128–251). No RVF vaccination was reported in any of the affected herds in the previous 24 months. The median number of adult female cattle kept by households with RVF cases was 5 (range 1–14). Seven of the affected households reported keeping goats in addition to cattle, with a median of 1 (range 0–30) adult female goat. No other livestock were kept by affected households.

Data on cattle grazing management were available for 12 (71%) of the 14 households with RVF cases. Of the households with these data, five (42%) managed cattle under a zero grazing system (permanently housed with fodder cut and brought to animals), three (25%) managed cattle under a tethered grazing system (unhoused but restrained by long rope and moved between grazing areas near the households) and four (33%) managed cattle under a herded grazing system (unhoused and moved between grazing areas by a herdsperson). One (8%) household reported using a mix of zero and herded grazing systems for cattle.

### RVFV genotypes identified

An 802-bp fragment of RVFV genome segment S sequence was obtained and analysed from three RVF cases. Positive cattle came from Moshi Rural (SEBI-051 and SEBI-079) and neighbouring Moshi Municipality (SEBI-094) Districts.

**Figure 3.**
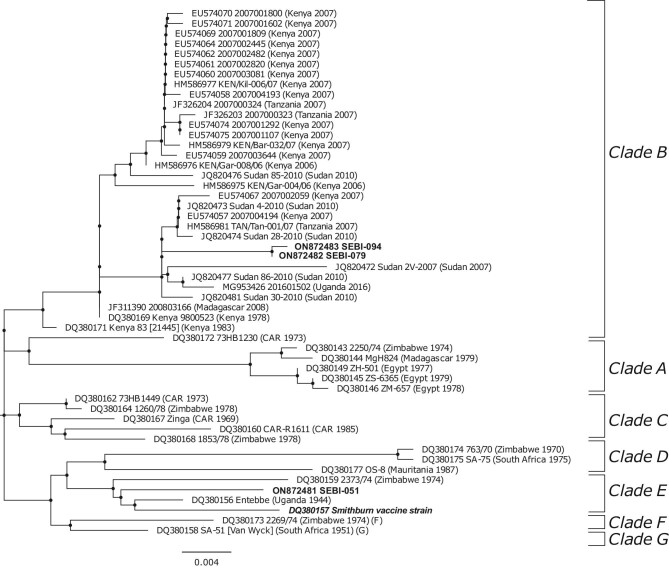
Molecular phylogenetic analysis of partial sequence (802 bp) of segment S of RVFV strains obtained from Tanzanian cattle, northern Tanzania, 2018. The tree with the highest log likelihood (−2414.72) is shown. Sequences are labelled with GenBank accession number and designated strain names with country and year of origin shown in parentheses. Sequences from this study are highlighted in bold and labelled with GenBank accession numbers and unique animal individual identifiers. Clades of viral lineages as designated by Bird et al. ^17^ are labelled by brackets. Sequence from the Smithburn vaccine strain is highlighted in bold italics.

Sequences obtained from SEBI-079 (GenBank accession no. ON872482) and SEBI-094 (GenBank accession no. ON872483) were highly similar (99.9%), with only a single nucleotide substitution between the two sequences. Based on phylogenetic analysis, these two sequences fell within the clade B haplotype^17^ alongside numerous sequences obtained from previous RVF outbreaks in livestock in Kenya, Tanzania and Sudan (Figure 3). In contrast, the third sequence (SEBI-051, ON872481) fell within the clade E haplotype and was more similar to sequences obtained from a South African outbreak in 2009 than sequences previously described in East Africa (Figure 3). Despite falling into the same clade as the Smithburn vaccine strain, a total of 11 nucleotide substitutions were detected between the Smithburn vaccine strain and the field sample SEBI-051 in the coding region of the partial sequence of the S segment used for phylogenetic analysis. In the translated amino acid sequences, seven variable sites were identified (amino acid numbers 16, 67, 180, 208, 222, 242 and 250) between the attenuated Smithburn vaccine strain and wild-type viruses within the same RVFV clade. When compared with the sequence obtained from SEBI-051, all nucleotide substitutions were non-synonymous mutations resulting in a change of amino acids at all sites. Therefore the virus obtained from SEBI-051 was considered to be a wild-type virus.

## Discussion

We describe an apparently small outbreak of RVF occurring among dairy cattle in a peri-urban area of northern Tanzania in 2018. Detection of livestock cases was accomplished through active, abortion-based sampling triggered by individual animal abortions rather than through a passive surveillance response to reports of unusually high levels of abortion. The retrospective detection of this previously unreported RVF outbreak provides evidence that RVF-associated livestock abortions are occurring more regularly in this endemic setting than would be expected on the basis of national surveillance alone. The occurrence of small-scale RVF outbreaks that go undetected by surveillance systems in endemic settings is not unexpected, but such events have rarely been described in detail.

The outbreak we describe has several notable features. First, all RVF cases were detected in animals reared in a peri-urban area. Transmission in urban and peri-urban areas is a key public health concern for many arboviruses^21^ but has been infrequently described for RVFV. Indeed, the WHO reports that there is no evidence for RVF outbreaks in urban areas,^22^ which could be taken to imply that RVF does not pose a threat to urban populations. In Tanzania, the peri-urban livestock sector is undergoing rapid expansion to meet the growing demand for milk and meat.^23^ Growing livestock populations within and surrounding high-density human population areas may pose new risks for zoonotic disease transmission,^24^ including for RVF.

These zoonotic risks are highlighted by the detection of RVFV nucleic acids in milk from multiple aborting dams sampled in this study. All RVF-associated abortions we identified were dairy cattle reared in an area that supplies the majority of milk to the town of Moshi.^25^ To our knowledge, the only published report of RVFV detection in milk was from experimentally infected animals in the 1950s.^26^ Our samples were preserved in a viricidal solution, so we were unable to assess the infectivity of milk from RVF cases, but milk-borne RVFV transmission is supported by literature-based reports of significant positive associations between RVFV seropositivity in people and a history of unboiled milk consumption.^27^ Few, if any, data exist on the effectiveness of heat treatment for inactivation of RVFV in milk and milk products. RVFV viability using culture-adapted virus has been detected following short periods of heat treatment at 56°C,^28^ raising concerns about the potential thermal stability of RVFV. In heat treatment experiments performed by our group using the RVFV-MP12 vaccine strain spiked in milk, we found no viral survival after incubation at 72°C for 15 min (further detail in the supplementary materials). Other authors report complete inactivation of RVFV after heating solutions containing the virus for 1 h at 60°C.^29^ These data suggest heat treatment is likely to reduce the risk of RVFV transmission via milk, but further work is needed to explore the effectiveness of standard milk pasteurisation and other milk preparation procedures on inactivation of the virus. Milk can be an important source of nutrition for food insecure households in RVF-endemic settings and interventions aimed at promoting milk safety should be carefully designed to avoid unintended consequences such as reduced consumption or inequitable access to safe products.^25^

A second notable feature of this outbreak is that despite its apparently limited spatial and temporal extent, we identified the circulation of viruses from two distinct lineages. The outbreak around Moshi occurred at the same time as outbreaks involving human and livestock populations were occurring in Kenya, Rwanda and Uganda.^30^ It is therefore possible that there were repeated virus introductions via livestock movements from neighbouring countries, which could explain the co-occurrence of these two viral haplotypes. This would also suggest that the Moshi outbreak was part of a more widespread, regional-level RVFV transmission rather than a small, isolated RVF outbreak arising autochthonously in northern Tanzania. Alternatively, the apparent viral diversity observed could be explained by repeated emergence from the low-level endemic viral cycling among livestock that has been described in Tanzania^2,5,6^ or spillover from unknown sylvatic cycles. It is worth noting that the town of Moshi sits on the edge of Kilimanjaro National Park and one of the wards in which cases were identified (Machame Mashiriki) directly borders this forested area. While limited data exist, it has been suggested that RVFV may circulate in unidentified sylvatic cycles in the forests of East Africa.^31^ Attempts to confirm and characterise these cycles in northern Tanzania would represent a valuable area for future research. At the time of writing, we are not aware of viral sequence data from the outbreaks in the wider East African region in 2018 that could help address questions around virus origin. These unanswered questions, and the diversity of viral strains we observed during this small outbreak, clearly demonstrate the value of genotyping during RVF oubreaks.^32^

It is also noteworthy that these two distinct viral lineages were identified in samples from only 3 of 14 RVF cases, raising the possibility of greater diversity than observed here. We performed genotyping on RT-qPCR products from placental and foetal samples, which tended to have the lowest Ct values (and therefore the highest concentration of viral RNA). Of the 12 available placental or foetal samples, insufficient sample was available for three after testing had been completed on the available aliquot at the University of Glasgow for the range of abortigenic pathogens evaluated as part of the wider research study^9^ (an additional aliquot has been retained in Tanzania). Among the six aliquots in which genotyping was not successful, two (SEBI-064 and SEBI-076) had Ct values in placental and/or foetal samples that were equivalent to the three samples in which we were able to amplify sufficient PCR product for genotyping (i.e. a Ct of ≤26). Although numbers are too small to draw firm conclusions, a hypothesis for this failure is primer mismatch with these viral haplotypes. The primers used in this study were adapted from an assay developed for working with the MP12 (lab modified) strain of RVFV rather than wild-type viruses. This may have resulted in a higher proportion of samples with amplification success of some haplotypes rather than others. Of note, a longer RT step was required for SEBI-051, which aligned with RVFV clade E, compared with SEBI-079 and SEBI-094, which aligned with RVFV clade B, which would support this hypothesis. A priority for future work will be to perform whole genome sequencing to overcome these potential issues with primer mismatch and to further enhance our understanding of the RVFV viral diversity associated with this outbreak.

We show that RVFV nucleic acids can be detected in a range of sample types collected within 72 h of cattle abortion. The number of available RVF cases was small and all sample types were not available for all animals, but placental material was most consistently RT-qPCR positive among the RVF cases, followed by swabs from the foetal surface. Encouragingly, vaginal swabs also appear to have good sensitivity for RVFV detection. Sampling directly from the dam has considerable advantages over collecting swabs or material from the foetus or placenta, which are frequently scavenged by dogs and wildlife or can become rapidly autolysed in hot climates. A surveillance system in which vaginal swabs are collected from recently aborted dams by livestock field officers or other trained people living in livestock-keeping communities and submitted for PCR-based testing at a regional laboratory, particularly during high-risk periods,^8^ would enable the early detection of RVF outbreaks, including small-scale outbreaks such as that described here. This could allow ring vaccination around an outbreak and the implementation of targeted livestock movement restrictions, reducing the risks of onward spread.^33^ Although there are likely to be many logistic and economic challenges with such a system, particularly given the historically low levels of investment in veterinary surveillance in many RVF-endemic countries,^34^ these costs and challenges may be offset if early detection of RVF cases and targeted interventions can contribute to preventing RVF outbreaks from becoming established as national and regional epidemics. In Tanzania, previous large-scale epidemics have resulted in widespread human illness and mortality, losses of large numbers of livestock and major impacts on the livestock sector through regional movement restrictions and slaughter bans.^4,35^

The small number of cases detected in this study, and the apparent geographic isolation of cases, suggest the occurrence of a small-scale RVF outbreak. However, there are important limitations to the prospective cohort study in which RVF cases were detected that create uncertainty about the true size of the outbreak we describe. In particular, there were substantial differences in the number of abortions investigated in each study ward, with reporting likely to be strongly biased by levels of engagement of community members with their LFO. A particular source of this bias that is reflected in our data is the large number of European dairy breeds and their crosses in the study sample and among cases. These animals are typically higher value than local breeds and can therefore be expected to receive higher levels of veterinary care. European-breed dairy cattle and their crosses are primarily reared in smallholder, peri-urban areas in northern Tanzania and are relatively rare across rural areas of the region.^10^ Smallholder dairy systems should therefore be considered to be overrepresented in our sample. Given this selection bias, we cannot rule out a larger RVF outbreak or multiple separate outbreaks occurring over a wider geographic area or longer time period. All RVF abortions were detected during a high-rainfall period in which multiple areas of northern Tanzania were considered to be at high risk for RVF outbreaks.^8,30^

## Conclusions

Testing of samples collected as part of a research study investigating the aetiology of livestock abortion allowed us to retrospectively identify a small outbreak of RVF occurring among livestock in Tanzania in 2018 that involved RVF viruses from two distinct lineages. Our data provide a rare example of the type of small-scale RVF outbreak that can occur below the surveillance detection threshold in endemic countries. The outbreak we describe occurred among cattle in the emerging dairy sector in the peri-urban area surrounding the town of Moshi. Importantly, we identified shedding of RVFV nucleic acids in milk from affected animals, supporting growing evidence for milk as a potential source of human RVFV infection, potentially including urban consumers. Promotion of milk safety measures, including pasteurisation and home boiling, may therefore contribute to reduced zoonotic RVF risk in endemic settings during high-risk periods. Our data suggest that RT-qPCR performed on vaginal swabs collected within 72 h of a bovine abortion has good sensitivity for the detection of RVFV nucleic acids from RVF abortion cases. This simple and low-cost sampling approach has the potential to be integrated into active abortion-based surveillance, potentially enabling early detection and response to RVF outbreaks.

## Supplementary Material

trac076_Supplemental_File

## Data Availability

The data underlying this article are available in the article and in its online supplementary material. The sequences generated by this study are available through NCBI GenBank (accession numbers ON872481–872483).
